# Unfolding of the Amyloid β-Peptide Central Helix: Mechanistic Insights from Molecular Dynamics Simulations

**DOI:** 10.1371/journal.pone.0017587

**Published:** 2011-03-07

**Authors:** Mika Ito, Jan Johansson, Roger Strömberg, Lennart Nilsson

**Affiliations:** 1 Department of Biosciences and Nutrition, Karolinska Institutet, Huddinge, Sweden; 2 Department of Anatomy, Physiology and Biochemistry, Swedish University of Agricultural Sciences, the Biomedical Centre, Uppsala, Sweden; 3 Department of Neurobiology, Care Sciences and Society (NVS), Karolinska Institutet Alzheimer Disease Research Center (KI-ADRC), Huddinge, Sweden; Kings College, London, United Kingdom

## Abstract

Alzheimer's disease (AD) pathogenesis is associated with formation of amyloid fibrils caused by polymerization of the amyloid β-peptide (Aβ), which is a process that requires unfolding of the native helical structure of Aβ. According to recent experimental studies, stabilization of the Aβ central helix is effective in preventing Aβ polymerization into toxic assemblies. To uncover the fundamental mechanism of unfolding of the Aβ central helix, we performed molecular dynamics simulations for wild-type (WT), V18A/F19A/F20A mutant (MA), and V18L/F19L/F20L mutant (ML) models of the Aβ central helix. It was quantitatively demonstrated that the stability of the α-helical conformation of both MA and ML is higher than that of WT, indicating that the α-helical propensity of the three nonpolar residues (18, 19, and 20) is the main factor for the stability of the whole Aβ central helix and that their hydrophobicity plays a secondary role. WT was found to completely unfold by a three-step mechanism: 1) loss of α-helical backbone hydrogen bonds, 2) strong interactions between nonpolar sidechains, and 3) strong interactions between polar sidechains. WT did not completely unfold in cases when any of the three steps was omitted. MA and ML did not completely unfold mainly due to the lack of the first step. This suggests that disturbances in any of the three steps would be effective in inhibiting the unfolding of the Aβ central helix. Our findings would pave the way for design of new drugs to prevent or retard AD.

## Introduction

Amyloid β-peptide (Aβ) was first isolated from meningeal vessels of Alzheimer's disease (AD) patients [1], and one year later, it was recognized as the main component of the neuritic (senile) plaques in AD patients' brain tissue [2]. The amyloid cascade hypothesis suggests that accumulation of Aβ in the brain is the primary influence driving AD pathogenesis [3], [4], [5]. According to this hypothesis, cleavage of the amyloid precursor protein at the membrane of a neuronal cell produces Aβ, subsequent aggregation and fibril formation of Aβ in extracellular fluid produce a neuritic plaque, and neuronal death is caused by toxicity of prefibrillar intermediates or mature fibrils, which eventually causes AD. Despite many efforts, fundamental and practical treatments and drugs for AD have not yet been found, even though several drugs to help mask the symptoms of AD are already being used.

A recent experimental study [6] reported that, although immunization with Aβ resulted in clearance of the neuritic plaques in AD patients, this did not prevent progressive neurodegeneration. This suggests that it may be too late to cure or prevent AD after the emergence of plaques. To find effective methods or drugs to prevent AD, the mechanism of Aβ fibril formation, which takes place before the emergence of plaques, should be clarified in detail.

In early experimental studies [7], [8], [9], [10] it was shown that Aβ consist of 39–42 amino acids, and that a short Aβ(16–20) peptide composed of the middle five residues (KLVFF) is capable of binding to full-length Aβ(1–40) [7]. By comparing the binding capabilities of many peptide sequences for Aβ(1–40), it was indicated that KLVFF is a minimum sequence for formation of the Aβ aggregate [7], [8]. In line with this, fluorescence-labeled KLVFF was found to specifically recognize immobilized KLVFF [9], [10].

Based on NMR structural data of Aβ in various environments, that is, membrane-water-medium-like environments [11], [12], [13] and extracellular-fluid-like environments [14], [15], and of an Aβ fibril [16], the middle region (15–24) of Aβ, which includes the Aβ recognition element (KLVFF), is considered to unfold after departing from the membrane to the extracellular fluid environment from an α-helix to a β-strand, accompanied by oligomerization and polymerization of the unfolded Aβ monomers into the form of β-sheets.

Circular dichroism spectroscopy and electron microscopy showed that the helical content of an α-helix/β-strand discordant region (residues 16–23) of Aβ(12–28) is increased by introducing V18A/F19A/F20A replacements or by adding the tripeptide KAD or phospho-L-serine, and this is associated with reduced fibril formation [17]. Thus, it was suggested that stabilization of the Aβ helical conformation would reduce Aβ fibril formation. Similarly, a study of lung surfactant protein C (SP-C), whose amyloid fibril formation is related to pulmonary alveolar proteinosis, showed that substitutions of leucines for valines in an α-helix/β-strand discordant region increase the helical content and reduce amyloid fibril formation [18]. In a recent experimental study it was shown that stabilization of the central α-helix of Aβ(1–40/42) by either of two classes of ligands, which were designed to bind and stabilize the 13–26 region in an α-helical conformation, counteracts Aβ polymerization into toxic assemblies, prevents Aβ induced reduction in hippocampal γ-oscillations, and increases longevity as well as decreases locomotor dysfunction in a *Drosophila* model of AD [19].

According to recent experimental studies [17], [19], inhibiting unfolding of the Aβ α-helix of the middle region (15–24) can be an effective strategy to repress Aβ aggregation and fibril formation, and is therefore a promising strategy for development of drugs to prevent or slow AD. In order to efficiently develop drugs according to this strategy, elucidation of the detailed molecular mechanism of the unfolding of the Aβ central helix is needed. However, this detailed mechanism remains obscure, due to the instability of the Aβ α-helix in the extracellular environment, and also because of experimental limitations in analyzing details of processes involving rapid structural changes of biomolecules. On the other hand, theoretical methods can overcome such limitations, and properties of model structures of short-lived species such as the Aβ α-helix in water can be studied using molecular dynamics (MD) methods. To date, many MD studies on the structure and dynamics of Aβ have been reported. These studies have mainly focused on oligomerization of unfolded Aβ [20], [21], [22], [23], [24], [25], [26], with a smaller number of studies of Aβ unfolding [27], [28], [29], [30].

One recent MD study of the unfolding of Aβ examined the stability of helical forms of Aβ(1–42) and analogues [27], and indicated that the stability of the α-helical conformation of the middle region of the V18A/F19A/F20A mutant Aβ is higher than that of the wild-type Aβ, in agreement with experimental data [17]. However, the detailed mechanisms for the unfolding of the α-helical conformation of Aβ and for the stabilization by V18A/F19A/F20A replacements remain unclear.

In the present study, the unfolding of the Aβ central helix (residues 15–24) was investigated by MD simulations starting from α-helical structures of Aβ(13–26). The region (15–24) of Aβ is of interest since the KLVFF motif included in this region is capable of binding to full-length Aβ [7], [8] and to the KLVFF motif itself [9], [10], and stabilization of this region in an α-helical conformation by mutations or by ligands counteracts Aβ polymerization into toxic assemblies [17], [19]. MD simulations were executed under aqueous-solution conditions, because it is known that the unfolding of the Aβ central helix occurs when Aβ is exposed to the extracellular fluid environment. In order to gain a better understanding of the molecular mechanism of the unfolding of the Aβ central helix, the wild-type (WT) Aβ(13–26) model was compared with mutant Aβ(13–26) models with alanine (MA) or leucine (ML) replacements at three nonpolar residues 18, 19, and 20. The effect of V18A/F19A/F20A replacements [17] on the stability of the Aβ central helix has been reported previously, but that of V18L/F19L/F20L has never been reported to date. Finding a simulation protocol that is able to unfold the WT helix, but not the mutants, will also give us a span where we can later on study, in detail, the interactions of helix stabilizing ligands [17], [19] with Aβ.

Experimental studies [31], [32], [33], [34] of peptides and proteins which ultimately form organized structures such as amyloid fibrils suggest that the aggregation propensity of such peptides and proteins is associated with simple physicochemical properties of individual amino acids, such as hydrophobicity, secondary structure (α-helical and β-sheet) propensity, and charge. We therefore examined the effects of replacements of the nonpolar residues with two types of nonpolar residues (A and L) on the stability of the Aβ central helix. The rank order of the biological hydrophobicity (determined by Hessa et al. [35] with membrane insertion) is A<V<F<L. The hydrophobicity of A and L thus brackets that of V and F. At the same time the rank order (determined by Kallberg et al. [18] with amino acid distributions in helices and β-strands using the Chou-Fasman method [36]) of the α-helical propensity is V<F<L<A and that of the β-strand propensity is just the opposite, A<L<F<V.

It has been suggested that aggregation of peptides and proteins is generally promoted by hydrophobicity as such [37], [38], because hydrophobic regions of peptides and proteins are known to be crucial for triggering the aggregation process and to form the core of fibrils [37], [38] including the Aβ fibril [16], [39]. However, because the initial process of Aβ fibril formation *in vivo* is the unfolding of the Aβ helix, this initial process may not necessarily have a dependence on hydrophobicity but could mainly be dependent on amino acid α-helical propensity. The whole process of Aβ fibril formation *in vivo* might then be highly influenced by the amino acid α-helical propensity.

As to general properties of peptide α-helices, it is considered that breaking of backbone O(*i*)-HN(*i*+4) hydrogen bonds is an early event in unfolding of peptide α-helices [40], [41], [42]. For this to be productive and lead to unfolding, it is also necessary to consider other factors that may contribute to the stability of the folded and unfolded states. Sidechain attractive interactions, or salt bridges, between residues on the same side of the helix (*i.e.*, from residue *i* to residue *i*+4 or *i*+8, approximately [43]) are assumed to enhance the stability of peptide α-helices [40], [41], [42]. To precisely elucidate a possible mechanism for the unfolding of the Aβ central helix, we should consider not only breaking of α-helical backbone hydrogen bonds but also other energetic factors such as sidechain interactions.

Through examination of the WT, MA, ML models, we tried to reveal whether the α-helical propensity is the sole determining factor or if the hydrophobicity affects the stability of the Aβ central helix. Furthermore, we present detailed mechanisms for the unfolding of the Aβ central helix and for the stabilization by the replacement of the three nonpolar residues.

## Methods

### Preparation of Systems

Experimental data [11], [12], [13] have shown that the middle region (15–24) of Aβ adopts an α-helical conformation in membrane-water-medium-like environments, and therefore initial model structures of Aβ(13–26), whose sequence is HHQKLVFFAEDVGS, were built in an α-helical conformation using the Insight II program (version 2000) [44]. Since Aβ(13–26) is a fragment of the full peptide, the N- and C-termini in our model were made neutral by capping with N-terminal acetyl and C-terminal amide groups, respectively. The structures of the mutant Aβ(13–26) models were built by modifying the WT Aβ(13–26) model with V18A/F19A/F20A or V18L/F19L/F20L replacements.

According to the NMR structure (entry 1HZ3 [14] in the Protein Data Bank [45]) of the unfolded Aβ in water at pH 5.7, all of the ionizable residues are in their charged states, where H13 and H14 are protonated. However, to take the pH dependence of histidine into consideration, two variants of the WT, MA, and ML models, in which both of H13 and H14 are protonated (WT^+^, MA^+^, and ML^+^) or deprotonated at the N_δ_ atoms (WT^0^, MA^0^, and ML^0^), were prepared. The total charge of the models with protonated histidines is +1*e*, and in the unprotonated case it is −1*e*. Each model was solvated in a rhombic dodecahedron water box filled with TIP3P [46] water molecules with a minimum solute-wall distance of 10 Å. Water molecules with the oxygen atom less than 2.2 Å from any heavy peptide atom were deleted, and each system was neutralized by adding 1 chloride or 1 sodium counterion.

### MD Simulations

All calculations were carried out using the CHARMM22/CMAP force field [47], [48], [49] with the CHARMM program [50], [51]. The SHAKE [52] algorithm was applied to fix all covalent bonds containing a hydrogen atom allowing a 2 fs timestep to be used in the integration of Newton's equations. The nonbonded (van der Waals and Coulomb) interaction energies and forces were smoothly shifted to zero at 12 Å using the atom-based force-shift method [53], [54], and the nonbonded list was constructed with a cutoff of 16 Å and was updated every time any atom moved by more than 2 Å since the last update. Before MD simulations were carried out, structures of the solvated systems were optimized by 500 steps of steepest descent energy minimization with a harmonic restraint of 20 kcal/mol/Å^2^ on Aβ followed by 1500 steps of adopted basis Newton-Raphson energy minimization without a harmonic restraint on Aβ. After the systems were heated up to 300, 330, or 360 K gradually for 50 ps, five or ten independent 20 ns MD simulations were carried out (Table 1) for a total of 1.2 µs of simulation to increase sampling [55]. The MD simulations were performed for the optimized systems under periodic boundary conditions at a constant pressure (1 atm) using the Langevin piston method [56] with piston mass 400 amu, collision frequency 20 ps^−1^ and bath temperature (300, 330, or 360 K). The average temperature was checked every 4 ps, and was found to remain within 5 K of the target temperature after the heating MD run. Fast table lookup routines for non-bonded interactions [57] were used to increase speed of the MD simulations. During the MD simulations, no harmonic restraints were imposed on any molecule in the systems, and coordinates were saved every 1 ps.

**Table 1:** Models and conditions of the MD simulations.

name of model	residues 18–20	state of histidines	temperature (K)	length (ns)/simulation	number of simulations
WT^+^	VFF	protonated	300	20	5
			330	20	5
			360	20	10
WT^0^	VFF	neutral	300	20	5
			330	20	5
			360	20	10
MA^+^	AAA	protonated	360	20	5
MA^0^	AAA	neutral	360	20	5
ML^+^	LLL	protonated	360	20	5
ML^0^	LLL	neutral	360	20	5

### Analyses

All analyses were carried out using every 10 ps of the trajectories after the heating time of the MD simulations, except as otherwise stated. Visualization of the structural change of the Aβ models during MD simulations was carried out by using the visual molecular dynamics (VMD) software (version 1.8.6) [58].

To examine the structural change of the Aβ models numerically, the root-mean-square deviation (RMSD) and radius of gyration (R_g_) were calculated for the middle region (15–24) of the Aβ models, thus large fluctuations of the RMSD and R_g_ due to the mobile N- and C-termini were eliminated. Before the RMSD measurements, overall rotation and translation were removed by least-squares superposition using coordinates of all heavy atoms of the initial energy-minimized structure obtained prior to the MD simulations. The RMSD was calculated for backbone heavy atoms against the initial energy-minimized coordinates and the R_g_ was calculated for all atoms along the MD simulation time.

To discriminate the type or the pattern of the Aβ structure, the number of α-helical backbone hydrogen bonds (αHBs) in the middle region (15–24) was calculated, using the criterion acceptor-hydrogen distance <2.4 Å to define the existence of a hydrogen bond [41]. The six αHBs in the middle region are numbered sequentially from 1 to 6 starting with the 15–19 residue pair.

To indicate the driving forces for the Aβ unfolding, energy profiles of the Aβ models were calculated, focusing in particular on the nonbonded interaction energies (van der Waals and Coulomb) between residues, separated into interaction energies between the seven polar residues (*E*_p-p_) and between the seven nonpolar residues (*E*_np-np_). The interaction energies between the polar residues and the nonpolar residues were not focused on, since they do not exhibit any notable changes during the simulations. For the calculations of *E*_p-p_ and *E*_np-np_, the interaction energy between one polar or nonpolar residue and the other six polar or nonpolar residues was calculated, and summation of the interaction energies of the seven polar or nonpolar residues was divided by two to avoid double counting of the interaction energy. In addition, *E*_np-np_ was analyzed in detail at the residue level to find which nonpolar residues mainly contribute to the nonpolar-nonpolar interactions.

## Results

### Aβ Unfolding

#### Stability of the α-Helical Conformation of WT

To examine at which temperature the unfolding of the WT peptide can be observed in the 20 ns MD simulations, five independent simulations at 300 K, five independent simulations at 330 K, and ten independent simulations at 360 K were performed for each WT model (WT+ and WT0).

**Figure 1:**
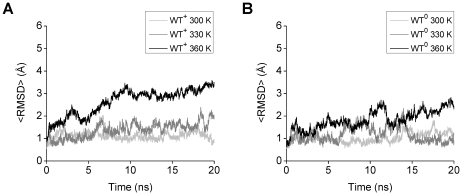
Mean RMSD (<RMSD>) of the middle region (15–24) of the WT Aβ models. The <RMSD> calculated for the WT^+^ (A) and WT^0^ (B) models are shown. The <RMSD> of each WT model at 300 K (light gray lines) or 330 K (dark gray lines) was calculated by using the five trajectories (trajectories 1–5), and that at 360 K (black lines) was calculated by using the ten trajectories (trajectories 1–10).

Similar tendencies were observed for WT^+^ and WT^0^ in the mean RMSD averaged over all trajectories (<RMSD>) (Fig. 1). The <RMSD> of both WT^+^ and WT^0^ at 360 K increased in the first 15 ns and leveled off in the last 5 ns, while at 300 K and 330 K the <RMSD> remained relatively small during 20 ns (Fig. 1). The fluctuations of the <RMSD> of both WT^+^ and WT^0^ at 360 K are small, particularly in the last 2 ns. The <RMSD> of both WT^+^ and WT^0^ at 360 K are on average more than 1.2 Å larger than at 300 K and 330 K in the last 2 ns (Fig. 1). By visual inspection of the structures, it was found that the WT peptide maintained its middle region (15–24) in the initial α-helical conformation during the whole 20 ns simulations in most of the trajectories at 300 K and at 330 K, but at 360 K the α-helical conformation was lost in several trajectories.

The average RMSD and the average number of αHBs of the last 2 ns at 360 K showed three types (A, B, and C) of behavior (Table 2). The seven trajectories in group A have relatively small (<2.0 Å) RMSD and 2 to 4 αHBs, suggesting that the peptide maintained the initial α-helical conformation during the whole simulations or refolded by the end of the simulations. The eight trajectories in group B have relatively large RMSD (≥2.0 Å) and 1 to 3 αHBs, indicating partial unfolding of the peptide. The five trajectories in group C have relatively large RMSD (≥4.0 Å) and no αHBs, suggesting complete unfolding of the peptide. Visual inspection of the structures confirmed that, by the end of the simulations at 360 K, the peptide refolded (though it partially unfolded during the simulations) in the seven A trajectories, partially unfolded in the eight B trajectories, and completely unfolded in the five C trajectories.

**Table 2:** Average RMSD (in Å) and average number of αHBs during the last 2 ns of 20 ns of the MD simulations calculated for WT at 360 K.

	average RMSD		average number of αHBs		group^a^	
trajectory	WT^+^	WT^0^	WT^+^	WT^0^	WT^+^	WT^0^
1	2.59	4.36	2.4	0.1	B	C
2	2.79	1.25	2.8	4.1	B	A
3	2.20	1.35	2.5	3.8	B	A
4	5.25	1.14	0.0	3.6	C	A
5	3.84	4.37	0.9	1.6	B	B
6	2.29	1.40	1.8	4.1	B	A
7	4.85	1.23	0.4	3.8	C	A
8	3.18	3.59	1.8	1.4	B	B
9	4.89	4.02	0.1	0.0	C	C
10	1.24	1.82	4.2	2.6	A	A
mean value^b^	3.31	2.45	1.7	2.5		
SD^c^	1.35	1.43	1.3	1.6		

a The trajectories are classified into three groups: A) RMSD<2.0 Å and 2≤αHBs≤6, B) RMSD≥2.0 Å and 1≤αHBs≤4, and C) RMSD≥4.0 Å and αHBs≈0.

b Mean values were calculated by using the ten average values obtained for each model.

c Standard deviation (SD) were calculated by using the ten average values obtained for each model.

#### Unfolding Mechanism of WT

Structural and energetic aspects of the WT trajectories were analyzed in detail, in order to understand how the Aβ central helix completely unfolds. One WT trajectory (WT+4) leading to complete unfolding is described in detail, followed by a summary for the other four trajectories (WT+7, WT+9, WT01, and WT09) that also displayed complete unfolding.

**Figure 2:**
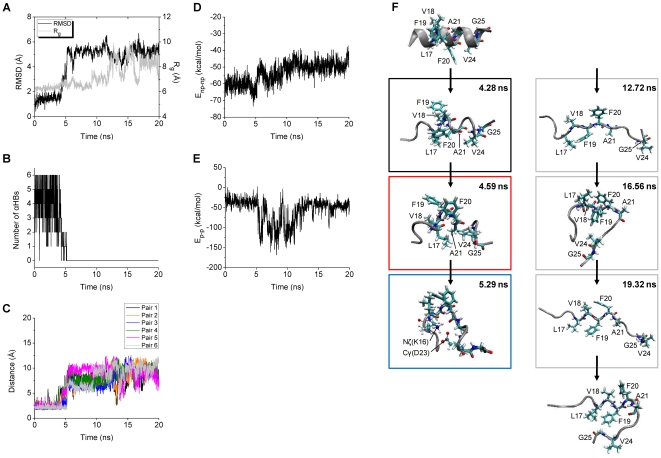
Structural and energetic changes of WT^+^4. The RMSD and R_g_ (A), the number of αHBs (B), and the backbone O-HN distances of the αHB pairs 1–6 (C) calculated for the middle region (15–24) of the Aβ model are shown. The nonbonded interaction energies including *E*_np-np_ (D) and *E*_p-p_ (E) are also shown. The structure obtained at 4.28 ns when the number of αHBs starts to decrease, that obtained at 4.59 ns with the *E*_np-np_ minimum (−73.58 kcal/mol), and that obtained at 5.29 ns with the notably low *E*_p-p_ (−116.13 kcal/mol) are displayed in the black, red, and blue boxes, respectively (F). The structures obtained at 12.72, 16.56, and 19.32 ns with relatively large (9.93 Å), small (6.48 Å), and large (9.65 Å) R_g_, respectively, are displayed from the top the bottom in the grey boxes. The initial energy-minimized structure and the structure obtained at 20.00 ns are also displayed at the top and the bottom, respectively. The positions of all the nonpolar residues (thick lines) and those of the polar residues (lines and balls) which are closely located are indicated.

In WT^+^4, a marked increase in RMSD at around 5 ns (from about 1.5 to 5.5 Å) is followed by an increase in R_g_ at around 12 ns (from about 7 to 9 Å) (Fig. 2A). Since RMSD was calculated for only backbone heavy atoms of the middle region while R_g_ was calculated for all atoms of the middle region, this shows that the middle region adopts a conformation with a stretched backbone and interacting sidechains from around 5 to 12 ns. After 12 ns, both RMSD and R_g_ are large, consistent with a fully extended conformation. The complete unfolding thus was triggered at around 5 ns in the WT^+^4 trajectory, and we inspected the time courses of several variables (number of αHBs, interaction energies between nonpolar (*E*_np-np_) and polar (*E*_p-p_) sidechains) of the trigger point (around 5 ns) to find changes in these variables that were of a larger magnitude than the high-frequency fluctuations.

Just before 5 ns the number of αHBs decreases from around 6 to 1 (Fig. 2B). All αHBs except for αHB1 are present almost constantly before 4.28 ns, but from 5.21 ns they are all broken until the end of the 20 ns simulation (Fig. 2C). The most favorable *E*_np-np_ (the deepest minimum in *E*_np-np_) occurs at 4.59 ns (Fig. 2D), close to the time of the large change in the number of αHBs. However, *E*_np-np_ at 4.28 ns when the number of αHBs starts to decrease is similar to *E*_np-np_ at the beginning of the simulation, which is around 10 kcal/mol less favorable than the *E*_np-np_ minimum. A quite favorable *E*_p-p_ occurs at 5.29 ns (Fig. 2E). The number of αHBs, *E*_np-np_, and *E*_p-p_, in this order, thus exhibit notable changes (Table 3).

**Table 3:** Timetable (in ns) of the three steps obtained for the WT complete unfolding processes at 360 K.

	step 1			step 2			step 3		
trajectory^a^	time	RMSD^b^	αHBs^c^	time	RMSD^b^	*E*_np-np_ d	time	RMSD^b^	*E*_p-p_ e
WT^+^4	4.28	1.47	3	4.59	3.01	−73.58	5.29	5.51	−116.13
WT^+^7	0.10	0.93	2	0.15	2.19	−66.32	1.36	4.26	−113.67
WT^+^9	15.21	2.85	0	15.53	2.93	−68.76	16.43	3.85	−70.04
WT^0^1	17.05	2.63	1	17.15	3.22	−74.15	18.15	3.99	−95.04
WT^0^9	9.09	1.54	1	9.20	2.18	−73.54	10.65	4.61	−87.82

a Only the trajectories in which the middle region of Aβ completely unfolded by 20 ns of the MD simulations are listed.

b RMSD (in Å) at each step are shown.

c Numbers of αHBs in the middle region counted at step 1 are shown.

d *E*_np-np_ (in kcal/mol) calculated at step 2 are shown.

e *E*_p-p_ (in kcal/mol) calculated at step 3 are shown.

The peptide conformations which were formed when the number of αHBs, *E*_np-np_, and *E*_p-p_ changed were analyzed in detail (Fig. 2F). At 4.28 ns the backbone of the middle region of the peptide is helical and straight, even though three out of the six αHBs are broken. At 4.59 ns the backbone of the middle regions is unwound, and the sidechains of the nonpolar residues L17, V18, F19, F20, A21, and V24 form a hydrophobic cluster. At 5.29 ns the backbone of the middle region is more unwound forming a bent conformation, with a salt bridge between residues K16 and D23 (the Nζ(K16)-Cγ(D23) distance is 3.36 Å *vs* 8.86 Å in the initial energy-minimized structure). The above structural data show that, in WT^+^4, after at least three out of the six αHBs were broken, the backbone was unwound by interactions between the sidechains of the nonpolar residues and further unwound by subsequent interactions between the sidechains of the polar residues.

Once the backbone is fully unwound and becomes more flexible, the middle region of the peptide changes between fully extended and compact conformations (Fig. 2F, right side). The total interaction energy between all residues calculated for the compact conformation is about 10 kcal/mol more favorable than the total energies calculated for the fully extended conformations. This indicates that in the absence of intermolecular interactions with other Aβ molecules or other molecular species, the completely unfolded middle region of Aβ can be stabilized by intramolecular interactions.

Altogether, in the WT^+^4 trajectory, the peptide completely unfolded through three steps: 1) loss of α-helical backbone hydrogen bonds, 2) strong interactions between sidechains of nonpolar residues, and 3) strong interactions between sidechains of polar residues. At the second step, a hydrophobic cluster composed of the sidechains of the nonpolar residues L17, V18, F19, F20, A21, and V24 allowing the backbone of the middle region to unwind, was formed, and facilitated the subsequent structural changes.

Applying the same detailed structural and energetic analyses, the WT peptide was also found to completely unfold through the same three steps in the other four trajectories (WT^+^7, WT^+^9, WT^0^1, and WT^0^9), regardless of the protonation states of the histidine residues (Table 3 and supplemental figures S1, S2, S3, and S4). The listed time (Table 3) of each step corresponds to the time at which the change in the number of αHBs, in *E*_np-np_, or in *E*_p-p_ is larger than the rapid fluctuations.

In all the five WT trajectories which exhibited complete unfolding, step 2 occurred within 0.5 ns after step 1, with step 3 following within 1.5 ns after step 2; the RMSD increased through the three steps, showing that the conformational change of the backbone of the middle region of the peptide increased as the process advanced (Table 3). At step 1 in the five trajectories, the backbone of the middle region of the peptide is still helical, even though three or more αHBs are broken. At step 2 in the five trajectories, the backbone of the middle region is unwound and sidechains of the nonpolar residues (L17, V18, F19, F20, A21, and V24) form a hydrophobic cluster (Fig. 2F, S1F, S2F, S3F, and S4F). Noteworthy is that the shapes of the hydrophobic clusters (Fig. 2F and S1F) formed at step 2 in two trajectories (WT^+^4 and WT^+^7) are quite similar. At step 3 in four trajectories (excluding WT^0^1), the N-terminal polar residues interact with the C-terminal polar residues, though at step 3 in one trajectory (WT^0^1), the N- and C-terminal polar residues interact locally with the other N- and C-terminal polar residues, respectively. It would be difficult for the N- and C-terminal polar residues to approach each other if the backbone did not become flexible before step 3, because the polar residues are separated by the central hydrophobic region (17–21) including the bulky nonpolar residues (L17, F19, F20). Thus, it seems that steps 1 and 2 are important in inducing the drastic conformational change of the WT peptide at step 3.

Additionally, the per-residue *E*_np-np_ at step 2 of the complete unfolding events in the five trajectories was analyzed (Table 4), to examine which nonpolar residues are important in forming a hydrophobic cluster allowing the backbone of the middle region to unwind. *E*_np-np_ of L17, V18, F19, F20, A21, and V24 are more favorable than *E*_np-np_ of G25 at step 2 in all five trajectories, and *E*_np-np_ of F19 and, in particular, F20 are favorable (<−20.0 kcal/mol). This indicates that L17, V18, A21, V24, and especially F19 and F20, contribute significantly to the disruption of the α-helical conformation of the WT peptide by forming the hydrophobic clusters.

**Table 4:** Per-residue *E*_np-np_ (in kcal/mol) at step 2 calculated for WT at 360 K.

		*E*_np-np_						
trajectory^a^	time	L17	V18	F19	F20	A21	V24	G25
WT^+^4	4.59	−21.64	−23.68	−22.65	−35.79	−18.93	−16.03	−8.43
WT^+^7	0.15	−16.43	−18.93	−23.41	−33.52	−21.74	−10.69	−7.91
WT^+^9	15.53	−14.33	−22.93	−20.15	−29.62	−20.21	−18.70	−11.59
WT^0^1	17.15	−18.37	−24.10	−26.16	−33.74	−20.85	−14.35	−10.74
WT^0^9	9.20	−21.75	−26.40	−26.52	−28.42	−21.85	−13.96	−8.18

a Only the trajectories in which the middle region of Aβ completely unfolded by 20 ns of the MD simulations are listed.

A three-step mechanism was thus proposed for the unfolding of the Aβ central helix based on similar structural and energetic features observed in the complete unfolding events of five WT trajectories. By the same detailed structural and energetic analyses, it was found that all of the partial-unfolding/refolding events in the WT trajectories lacked at least one of the steps of the three-step mechanism, indicating that all the three steps are necessary for the complete unfolding of the Aβ central helix.

### Effects of Alanine or Leucine Replacements on Aβ Unfolding

#### Stability of the α-Helical Conformation of MA and ML

**Figure 3:**
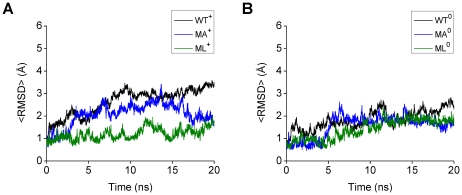
Mean RMSD (<RMSD>) of the middle region (15–24) of the WT, MA, and MT Aβ models. The <RMSD> calculated for the WT^+^, MA^+^, and ML^+^ models (A), and the WT^0^, MA^0^, and ML^0^ models (B) at 360 K are shown. The <RMSD> of each WT model (black lines) was calculated by using the ten trajectories (trajectories 1–10), and those of each MA (blue lines) or ML (green lines) model was calculated by using the five trajectories (trajectories 1–5).

MD simulations of the V18A/F19A/F20A (MA) and V18L/F19L/F20L (ML) models of Aβ(13–26) were carried out at 360 K. Five independent MD simulations were performed for each mutant Aβ model (Table 1). In comparison to WT, similar tendencies were observed for MA and ML in the mean RMSD averaged over all trajectories (<RMSD>) (Fig. 3). In the first 2 ns, the <RMSD> of MA and ML are almost constant around 1 Å, while for of WT there is an increase to 2 Å. The <RMSD> of MA and ML are smaller than those for WT in the last 2 ns (Fig. 3), showing that the conformations of the MA and ML peptides did not change so much by 20 ns at 360 K as the conformation of the WT peptide did under the same conditions.

Similar tendencies were observed for MA and ML in the average RMSD and in the average number of αHBs during the last 2 ns (Tables 5 and 6), in comparison to WT (Table 2). For the MA and ML trajectories, the RMSD is 0.7 to 1.8 Å smaller on average, and the number of αHBs is on average 1 to 2 larger on average, compared to the WT trajectories. Together with visual inspection of the structures, it was found that the MA and ML peptides maintained the initial α-helical conformations during the whole simulations or refolded by the end of the simulations in the seven MA and seven ML trajectories in the group A, and that they partially unfolded in the remaining three MA and three ML trajectories in the group B. Furthermore, for none of the MA and ML trajectories the average number of αHBs is smaller than 1, showing that neither of the MA and ML peptides completely unfolded in any of the ten MA trajectories or the ten ML trajectories by the end of the simulations, while the WT peptide completely unfolded in five out of the twenty WT trajectories. Taken together this indicates that the stability of the α-helical conformations of both the MA and ML peptides is higher than that of the WT peptide, regardless of the protonation states of the histidine residues.

**Table 5:** Average RMSD (in Å) and average number of αHBs during the last 2 ns of 20 ns of the MD simulations calculated for MA at 360 K.

	average RMSD		average number of αHBs		group^a^	
trajectory	MA^+^	MA^0^	MA^+^	MA^0^	MA^+^	MA^0^
1	0.74	1.24	4.4	4.3	A	A
2	1.03	1.23	4.1	4.5	A	A
3	4.72	4.03	1.9	1.1	B	B
4	0.83	0.64	4.2	4.4	A	A
5	2.23	0.77	3.6	4.2	B	A
mean value^b^	1.91	1.58	3.6	3.7		
SD^c^	1.68	1.39	1.0	1.5		

a The trajectories are classified into three groups: A) RMSD<2.0 Å and 2≤αHBs≤6, B) RMSD≥2.0 Å and 1≤αHBs≤4, and C) RMSD≥4.0 Å and αHBs≈0.

b Mean values were calculated by using the five average values obtained for each model.

c Standard deviation (SD) were calculated by using the five average values obtained for each model.

**Table 6:** Average RMSD (in Å) and average number of αHBs during the last 2 ns of 20 ns of the MD simulations calculated for ML at 360 K.

	average RMSD		average number of αHBs		group^a^	
trajectory	ML^+^	ML^0^	ML^+^	ML^0^	ML^+^	ML^0^
1	2.97	0.69	1.8	4.3	B	A
2	0.85	1.39	4.6	4.3	A	A
3	1.27	2.46	4.2	2.3	A	B
4	1.25	3.18	4.0	1.1	A	B
5	1.10	1.24	4.5	4.3	A	A
mean value^b^	1.49	1.79	3.8	3.2		
SD^c^	0.84	1.01	1.1	1.5		

a The trajectories are classified into three groups: A) RMSD<2.0 Å and 2≤αHBs≤6, B) RMSD≥2.0 Å and 1≤αHBs≤4, and C) RMSD≥4.0 Å and αHBs≈0.

b Mean values were calculated by using the five average values obtained for each model.

c Standard deviation (SD) were calculated by using the five average values obtained for each model.

The V18A/F19A/F20A replacements can stabilize the α-helical conformation of the middle region of Aβ, in agreement with experimental data for Aβ(12–28) [17] and with results of previous, shorter, MD simulations for the WT and MA models of full-length Aβ(1–42) [27]. V18L/F19L/F20L replacements can also stabilize the α-helical conformation of the middle region of Aβ, similarly to results for the middle region of SP-C [18]. It is noteworthy that the stability of the α-helical conformations of both the MA and ML peptides is higher than that of WT. This tendency is consistent with a higher α-helical propensity [18] for the three nonpolar residues (18, 19, and 20) of MA and ML than for the corresponding residues of WT. Thus, the stability of the Aβ central helix is strongly affected by the α-helical propensity of the three nonpolar residues (18, 19, and 20), whereas there is little effect of hydrophobicity, as such, for loss of helicity.

**Figure 4:**
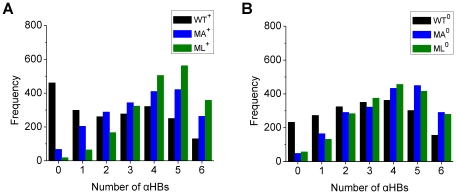
Average frequencies of the appearance of the structures with *n* αHBs (*n* = 0–6) in the middle region (15–24) of the WT, MA, and ML Aβ models. The average frequencies calculated for the WT^+^, MA^+^, and ML^+^ models (A) and the WT^0^, MA^0^, and ML^0^ models (B) at 360 K are shown. The average frequencies of each WT model (black bars) were calculated by using the ten trajectories (trajectories 1–10), and those of each MA (blue bars) or ML (green bars) model were calculated by using the five trajectories (trajectories 1–5).

In addition, the distribution of the number of αHBs was analyzed using the full 20 ns of all simulations (Fig. 4). The distribution is shifted towards higher numbers for MA and ML compared to WT, and in particular, the frequency of no αHBs is much lower for MA and ML. The frequency of no αHBs of MA and ML is one-fourth or lower than one-fourth of that of WT. In contrast, the frequency of six αHBs of MA and ML is two to three times as high as that of WT. From the distribution of the number of αHBs, it seems that the first step (sufficient loss of αHBs) of the three-step mechanism would be more difficult for the MA and ML peptides than for the WT peptide, resulting in the stabilization of the α-helical conformations of the MA and ML peptides.

#### Structural and Energetic Features of MA and ML

The structural and energetic aspects of the MA and ML trajectories were analyzed in detail, in order to understand how V18A/F19A/F20A and V18L/F19L/F20L replacements stabilize the Aβ central helix. Details for one MA (MA+2) trajectory and one ML (ML+3) trajectory are described below.

**Figure 5:**
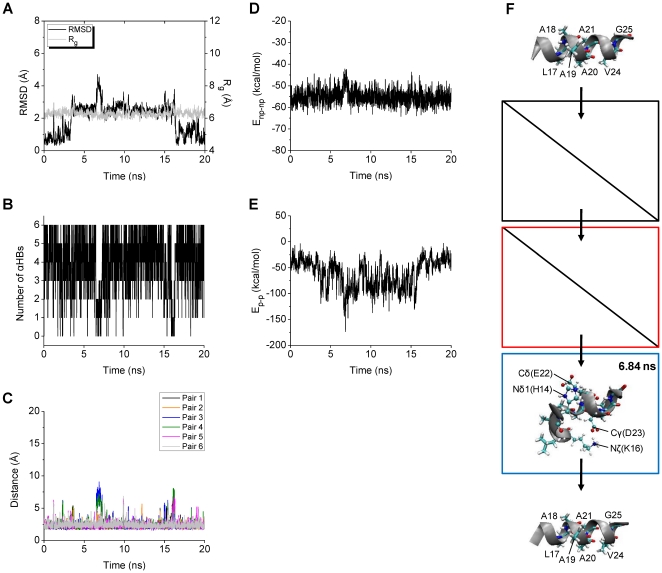
Structural and energetic changes of MA^+^2. The RMSD and R_g_ (A), the number of αHBs (B), and the backbone O-HN distances of the αHB pairs 1–6 (C) calculated for the middle region (15–24) of the mutant Aβ model are shown. The nonbonded interaction energies including *E*_np-np_ (D) and *E*_p-p_ (E) are also shown. The structure of MA^+^2 obtained at 6.84 ns with the *E*_p-p_ minimum (−173.43 kcal/mol) is displayed in the blue box (F). The initial energy-minimized structure and the structure obtained at 20.00 ns are also displayed at the top and the bottom, respectively. The positions of all the nonpolar residues (thick lines) and those of the polar residues (lines and balls) which are closely located are indicated.

In MA^+^2, RMSD is relatively large from about 3 to 17 ns, and a marked increase in RMSD is observed at around 7 ns, though Rg is almost constant during the whole simulation (Fig. 5A). The six αHBs are kept almost constantly during the whole simulation (Fig. 5B and 5C). *E*_np-np_ is almost constant (Fig. 5D) and a hydrophobic cluster was not observed during the whole simulation, suggesting that the sidechains of the three replaced nonpolar residues cannot reach those of other nonpolar residues to form a hydrophobic cluster. *E*_p-p_ is low from about 3 to 17 ns when RMSD is large, and a notable change in *E*_p-p_ is observed at around 7 ns when the marked increase in RMSD occurs (Fig. 5E). At 6.84 ns when *E*_p-p_ is minimum in MA^+^2, salt bridges H14-E22 and K16-D23 are formed (Fig. 5F). Although the backbone of residues 15–19 is unwound by the interactions between the sidechains of the polar residues, the backbone of residues 20–24 is still helical at 6.84 ns. The α-helical conformation of the middle region of the MA peptide is fully reconstructed at the end of the simulation in MA^+^2 (Fig. 5F).

**Figure 6:**
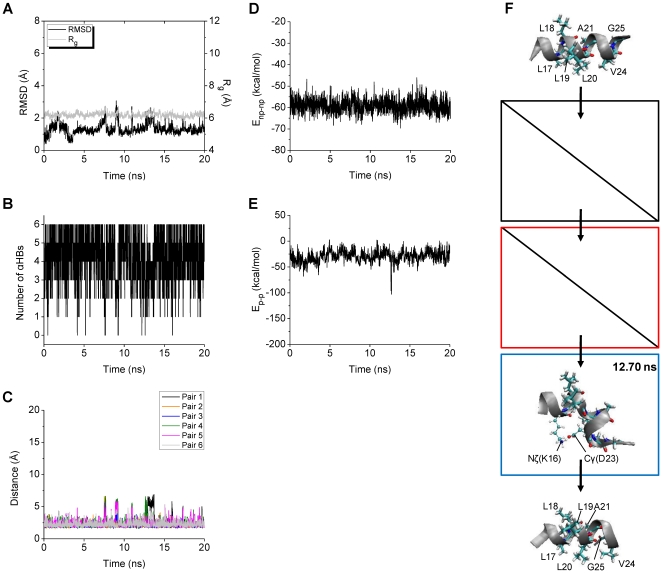
Structural and energetic changes of ML^+^3. The RMSD and R_g_ (A), the number of αHBs (B), and the backbone O-HN distances of the αHB pairs 1–6 (C) calculated for the middle region (15–24) of the mutant Aβ model are shown. The nonbonded interaction energies including *E*_np-np_ (D) and *E*_p-p_ (E) are also shown. The structure of ML^+^3 obtained at 12.70 ns with the *E*_p-p_ minimum (−102.95 kcal/mol) is displayed in the blue box (F). The initial energy-minimized structure and the structure obtained at 20.00 ns are also displayed at the top and the bottom, respectively. The positions of all the nonpolar residues (thick lines) and those of the polar residues (lines and balls) which are closely located are indicated.

In ML^+^3, several small increases in RMSD are observed before 15 ns, though Rg is almost constant during the whole simulation (Fig. 6A). The six αHBs are kept almost constantly during the whole simulation (Fig. 6B and 6C). *E*_np-np_ is almost constant (Fig. 6D) and a hydrophobic cluster was not observed during the whole simulation, suggesting that the sidechains of the three leucine residues cannot form a hydrophobic cluster, while the peptide is in helical conformation. Since there is a higher tendency, than for the wild type, to retain αHBs there is also not enough loss of αHBs to form hydrophobic clusters after such an event. A notable change in *E*_p-p_ is observed at around 13 ns when one of the increases in RMSD occurs (Fig. 6E). At 12.70 ns when *E*_p-p_ is minimum in ML^+^3, a salt bridge K16-D23 is formed (Fig. 6F). Although the backbone of the middle region of the peptide is bent by the interactions between the sidechains of the polar residues, it is still helical with a kink at residue 20 at 12.70 ns. The α-helical conformation of the middle region of the ML peptide is fully reconstructed at the end of the simulation in ML^+^3 (Fig. 6F).

To summarize, although the backbones of the middle regions of the MA and ML peptides were, temporarily, partially unwound by the interactions between the sidechains of the polar residues, they were not unwound so much as the backbone of the WT peptide in the complete unfolding events was, because all the αHBs were present almost constantly during the whole simulations in MA^+^2 and ML^+^3. This indicates that primarily the lack of step 1 of the three-step mechanism resulted in the stabilization of the α-helices in both MA^+^2 and ML^+^3. This lack of step 1 was also found in the other six MA and five ML trajectories in which α-helical conformations of the MA and ML peptides were maintained during the whole simulations or were reconstructed by the end of the simulations.

## Discussion

The unfolding process of the Aβ central helix (residues 15–24), which is a prerequisite for the amyloid fibril formation in AD, was investigated by MD simulations of wild-type (WT) Aβ(13–26) and two mutant forms with alanine (MA) or leucine (ML) substitutions at the three nonpolar residues (18, 19, and 20). As indicated mainly by the backbone RMSD *vs* the initial structure and the existence of αHBs, the WT peptide unfolded (completely or partially) to a large extent in simulations at 360 K (the temperature used to achieve unfolding in the 20 ns MD trajectories), whereas the MA and ML peptides essentially remained stable α-helices, indicating that the α-helical propensity of the three nonpolar residues (18, 19, and 20) is the dominating factor for the stability of the Aβ central helix. While there is little effect of the hydrophobicity on loss of helicity (there is also slight tendency for this when comparing the data for the MA and ML peptides in Figures 3 and 4), this can play a role in events taking place after initial unwinding of the helix. If residues have a high helical propensity hydrophobicity can even stabilize helices further, provided there is interaction between them.

That helical propensity is the main factor for loss of helicity is consistent with previous experimental data for Aβ(12–28) [17] and previous simulation data for full-length Aβ(1–42) [27] and also similar to experimental results for the middle region of SP-C [18]. The presented simulations thus further substantiate the importance of amino acid α-helical propensity in predicting sequences of Aβ-analogous or Aβ-variant peptides which may form amyloid fibrils *in vivo*.

Based on detailed structural and energetic analyses of the unfolding events observed in the WT trajectories, we suggest that complete unfolding occurs via a three-step mechanism, where the subsequent steps are dependent on that step 1 is completed: 1) sufficient loss of α-helical backbone hydrogen bonds, 2) strong interactions between nonpolar sidechains, and 3) strong interactions between polar sidechains. We identified a hydrophobic cluster composed of the sidechains of L17, V18, F19, F20, A21, and V24, that allowed the backbone of the middle region to unwind, at the second step. We found that especially residues F19 and F20 contribute significantly to the stabilization of the hydrophobic cluster. We consider that this hydrophobic cluster facilitate the transition to the third step with interactions between polar residues which are separated by the central hydrophobic region (17–21). The Aβ central helix did not completely unfold in cases when any of the three steps was missing. Thus, we suggest that the complete unfolding of this helix may be inhibited not only by preventing breakage of α-helical backbone hydrogen bonds (the first step) but also by disturbing interactions between nonpolar sidechains (the second step) or between polar sidechains (the third step).

In addition, the properties of the MA and ML peptides were compared to those of the WT peptide to examine the effects of alanine and leucine replacements on the Aβ unfolding mechanism. For both MA and ML the structures which possess all αHBs in the middle region appeared two to three times as often as for WT during the whole simulations, suggesting that the first step of the three-step mechanism would be more difficult for the MA and ML peptides than for the WT peptide. By detailed structural and energetic analyses of the MA and ML trajectories, we confirmed that the alanine and leucine replacements are effective in preventing the first step, associated with the higher α-helical propensity of these residues, to inhibit the unfolding of the Aβ central helix.

The quantitative assessments made from our MD simulations are in good agreement with available experimental data for the alanine mutant, which lends support to our findings for the leucine mutant as well as for the proposed three-step unfolding mechanism.

## Supporting Information

Figure S1 **Structural and energetic changes of WT^+^7.** The RMSD and R_g_ (A), the number of αHBs (B), and the backbone O-HN distances of the αHB pairs 1–6 (C) calculated for the middle region (15–24) of the Aβ model are shown. The nonbonded interaction energies including *E*_np-np_ (D) and *E*_p-p_ (E) are also shown. The structure obtained at 0.10 ns when the number of αHBs starts to decrease, that obtained at 0.15 ns with the *E*_np-np_ minimum (−66.32 kcal/mol), and that obtained at 1.36 ns with the notably low *E*_p-p_ (−113.67 kcal/mol) are displayed in the black, red, and blue boxes, respectively (F). The structures obtained at 2.11, 5.90, and 10.13 ns with relatively large (9.37 Å), small (6.48 Å), and large (9.69 Å) R_g_, respectively, are displayed from the top the bottom in the grey boxes. The initial energy-minimized structure and the structure obtained at 20.00 ns are also displayed at the top and the bottom, respectively. The positions of all the nonpolar residues (thick lines) and those of the polar residues (lines and balls) which are closely located are indicated.
(TIF)

Figure S2 **Structural and energetic changes of WT^+^9.** The RMSD and R_g_ (A), the number of αHBs (B), and the backbone O-HN distances of the αHB pairs 1–6 (C) calculated for the middle region (15–24) of the Aβ model are shown. The nonbonded interaction energies including *E*_np-np_ (D) and *E*_p-p_ (E) are also shown. The structure obtained at 15.21 ns when the number of αHBs starts to decrease, that obtained at 15.53 ns with the notably low *E*_np-np_ (−68.76 kcal/mol), and that obtained at 16.43 ns with the notably low *E*_p-p_ (−70.04 kcal/mol) are displayed in the black, red, and blue boxes, respectively (F). The structures obtained at 17.72, 18.32, and 19.07 ns with relatively large (8.64 Å), small (6.17 Å), and large (9.09 Å) R_g_, respectively, are displayed from the top the bottom in the grey boxes. The initial energy-minimized structure and the structure obtained at 20.00 ns are also displayed at the top and the bottom, respectively. The positions of all the nonpolar residues (thick lines) and those of the polar residues (lines and balls) which are closely located are indicated.
(TIF)

Figure S3 **Structural and energetic changes of WT^0^1.** The RMSD and R_g_ (A), the number of αHBs (B), and the backbone O-HN distances of the αHB pairs 1–6 (C) calculated for the middle region (15–24) of the Aβ model are shown. The nonbonded interaction energies including *E*_np-np_ (D) and *E*_p-p_ (E) are also shown. The structure obtained at 17.05 ns when the number of αHBs starts to decrease, that obtained at 17.15 ns with the *E*_np-np_ minimum (−74.15 kcal/mol), and that obtained at 18.15 ns with the notably low *E*_p-p_ (−95.04 kcal/mol) are displayed in the black, red, and blue boxes, respectively (F). The structures obtained at 18.75, 19.16, and 19.64 ns with relatively large (8.70 Å), small (6.78 Å), and large (8.94 Å) R_g_, respectively, are displayed from the top the bottom in the grey boxes. The initial energy-minimized structure and the structure obtained at 20.00 ns are also displayed at the top and the bottom, respectively. The positions of all the nonpolar residues (thick lines) and those of the polar residues (lines and balls) which are closely located are indicated.
(TIF)

Figure S4 **Structural and energetic changes of WT^0^9.** The RMSD and R_g_ (A), the number of αHBs (B), and the backbone O-HN distances of the αHB pairs 1–6 (C) calculated for the middle region (15–24) of the Aβ model are shown. The nonbonded interaction energies including *E*_np-np_ (D) and *E*_p-p_ (E) are also shown. The structure obtained at 9.09 ns when the number of αHBs starts to decrease, that obtained at 9.20 ns with the *E*_np-np_ minimum (−73.54 kcal/mol), and that obtained at 10.65 ns with the notably low *E*_p-p_ (−87.82 kcal/mol) are displayed in the black, red, and blue boxes, respectively (F). The structures obtained at 12.90, 16.60, and 17.22 ns with relatively large (9.19 Å), small (6.42 Å), and large (8.96 Å) R_g_, respectively, are displayed from the top the bottom in the grey boxes. The initial energy-minimized structure and the structure obtained at 20.00 ns are also displayed at the top and the bottom, respectively. The positions of all the nonpolar residues (thick lines) and those of the polar residues (lines and balls) which are closely located are indicated.
(TIF)
