# Supplementary figures and images for: Unfolding of the Amyloid β-Peptide Central Helix: Mechanistic Insights from Molecular Dynamics Simulations
Source: PLoS One. 2011 Mar 7;6(3):e17587. doi: 10.1371/journal.pone.0017587 (PMC3049775; doi:10.1371/journal.pone.0017587)

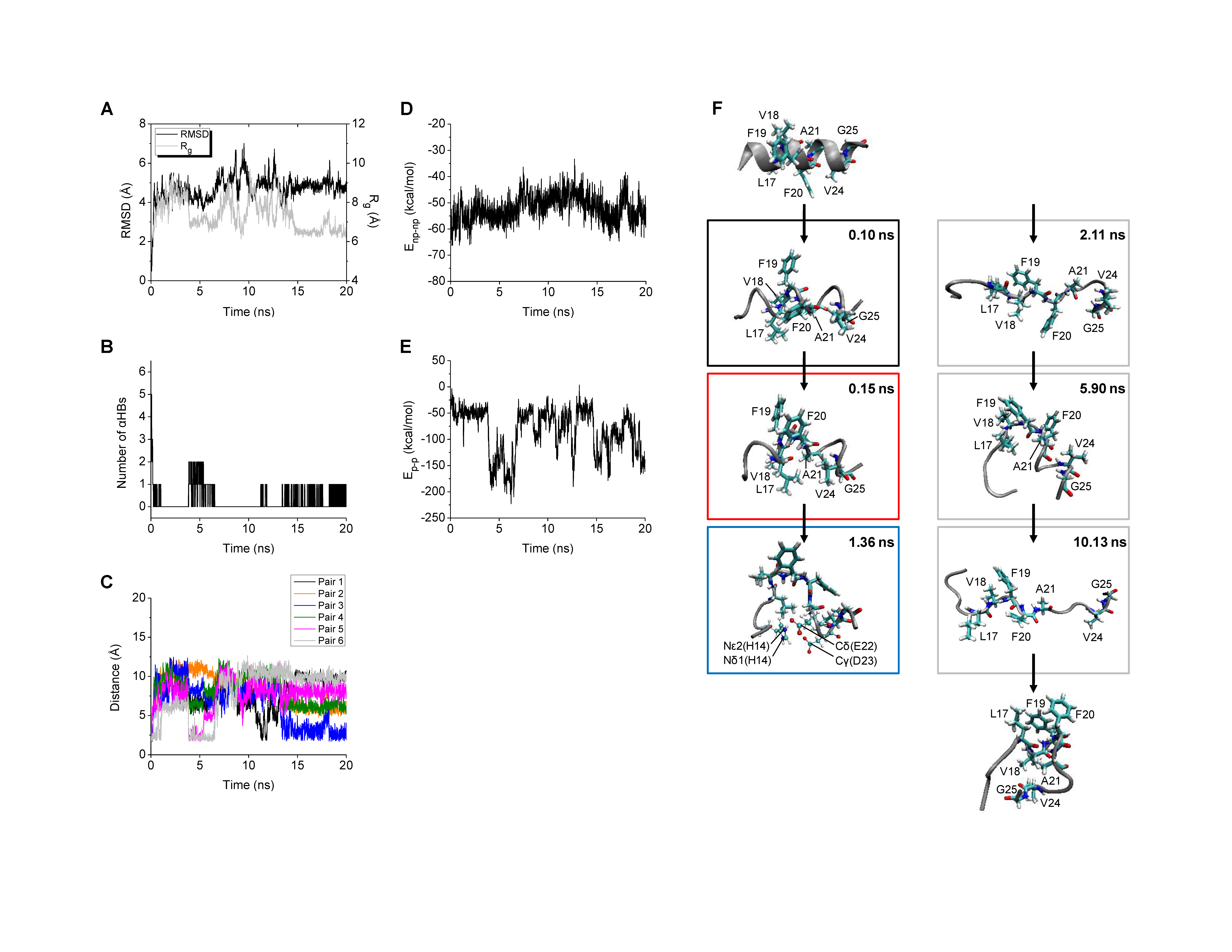

Supplement: Figure S1 — Structural and energetic changes of WT+7. The RMSD and Rg (A), the number of αHBs (B), and the backbone O-HN distances of the αHB pairs 1–6 (C) calculated for the middle region (15–24) of the Aβ model are shown. The nonbonded interaction energies including E np-np (D) and E p-p (E) are also shown. The structure obtained at 0.10 ns when the number of αHBs starts to decrease, that obtained at 0.15 ns with the E np-np minimum (−66.32 kcal/mol), and that obtained at 1.36 ns with the notably low E p-p (−113.67 kcal/mol) are displayed in the black, red, and blue boxes, respectively (F). The structures obtained at 2.11, 5.90, and 10.13 ns with relatively large (9.37 Å), small (6.48 Å), and large (9.69 Å) Rg, respectively, are displayed from the top the bottom in the grey boxes. The initial energy-minimized structure and the structure obtained at 20.00 ns are also displayed at the top and the bottom, respectively. The positions of all the nonpolar residues (thick lines) and those of the polar residues (lines and balls) which are closely located are indicated. (TIF) [file pone.0017587.s001.tif]

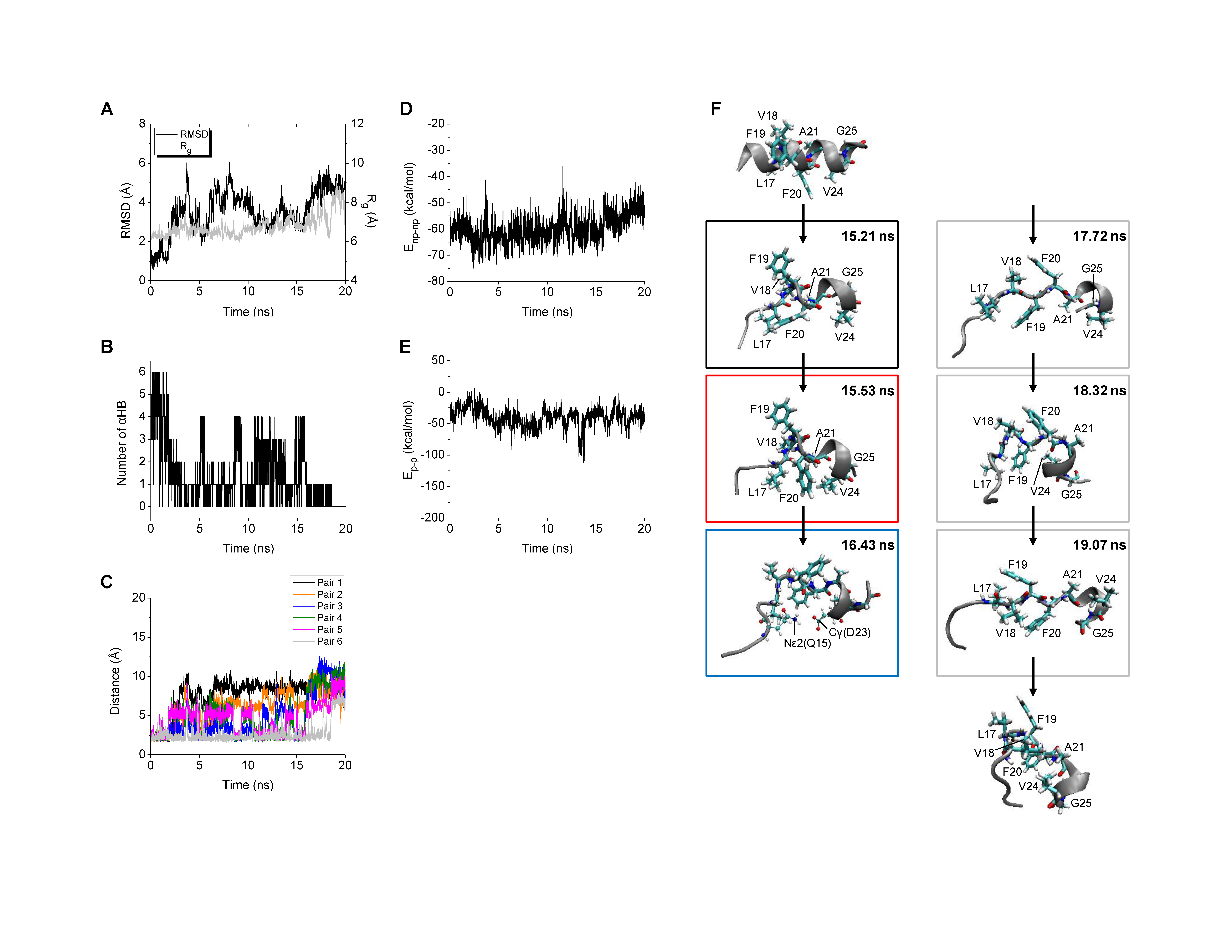

Supplement: Figure S2 — Structural and energetic changes of WT+9. The RMSD and Rg (A), the number of αHBs (B), and the backbone O-HN distances of the αHB pairs 1–6 (C) calculated for the middle region (15–24) of the Aβ model are shown. The nonbonded interaction energies including E np-np (D) and E p-p (E) are also shown. The structure obtained at 15.21 ns when the number of αHBs starts to decrease, that obtained at 15.53 ns with the notably low E np-np (−68.76 kcal/mol), and that obtained at 16.43 ns with the notably low E p-p (−70.04 kcal/mol) are displayed in the black, red, and blue boxes, respectively (F). The structures obtained at 17.72, 18.32, and 19.07 ns with relatively large (8.64 Å), small (6.17 Å), and large (9.09 Å) Rg, respectively, are displayed from the top the bottom in the grey boxes. The initial energy-minimized structure and the structure obtained at 20.00 ns are also displayed at the top and the bottom, respectively. The positions of all the nonpolar residues (thick lines) and those of the polar residues (lines and balls) which are closely located are indicated. (TIF) [file pone.0017587.s002.tif]

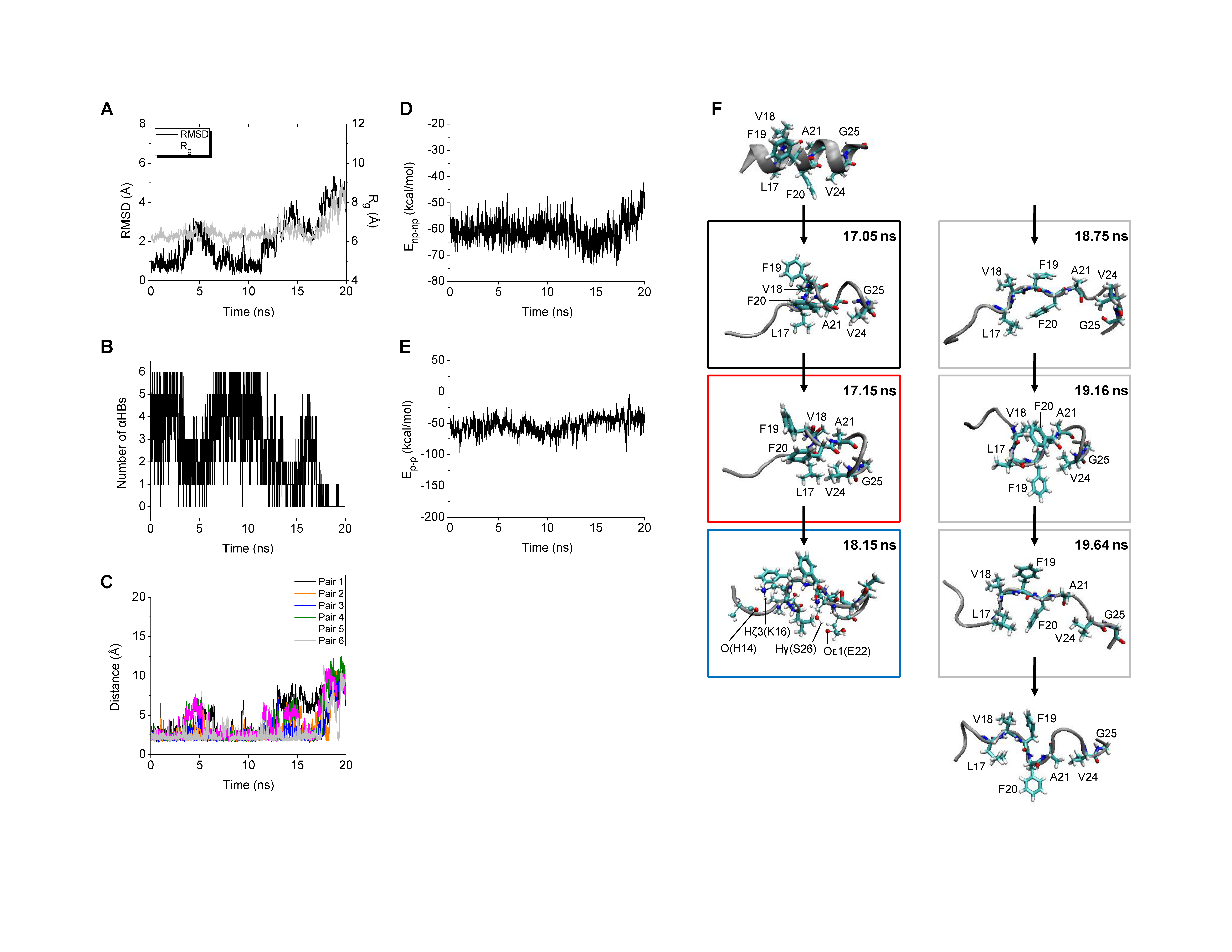

Supplement: Figure S3 — Structural and energetic changes of WT01. The RMSD and Rg (A), the number of αHBs (B), and the backbone O-HN distances of the αHB pairs 1–6 (C) calculated for the middle region (15–24) of the Aβ model are shown. The nonbonded interaction energies including E np-np (D) and E p-p (E) are also shown. The structure obtained at 17.05 ns when the number of αHBs starts to decrease, that obtained at 17.15 ns with the E np-np minimum (−74.15 kcal/mol), and that obtained at 18.15 ns with the notably low E p-p (−95.04 kcal/mol) are displayed in the black, red, and blue boxes, respectively (F). The structures obtained at 18.75, 19.16, and 19.64 ns with relatively large (8.70 Å), small (6.78 Å), and large (8.94 Å) Rg, respectively, are displayed from the top the bottom in the grey boxes. The initial energy-minimized structure and the structure obtained at 20.00 ns are also displayed at the top and the bottom, respectively. The positions of all the nonpolar residues (thick lines) and those of the polar residues (lines and balls) which are closely located are indicated. (TIF) [file pone.0017587.s003.tif]

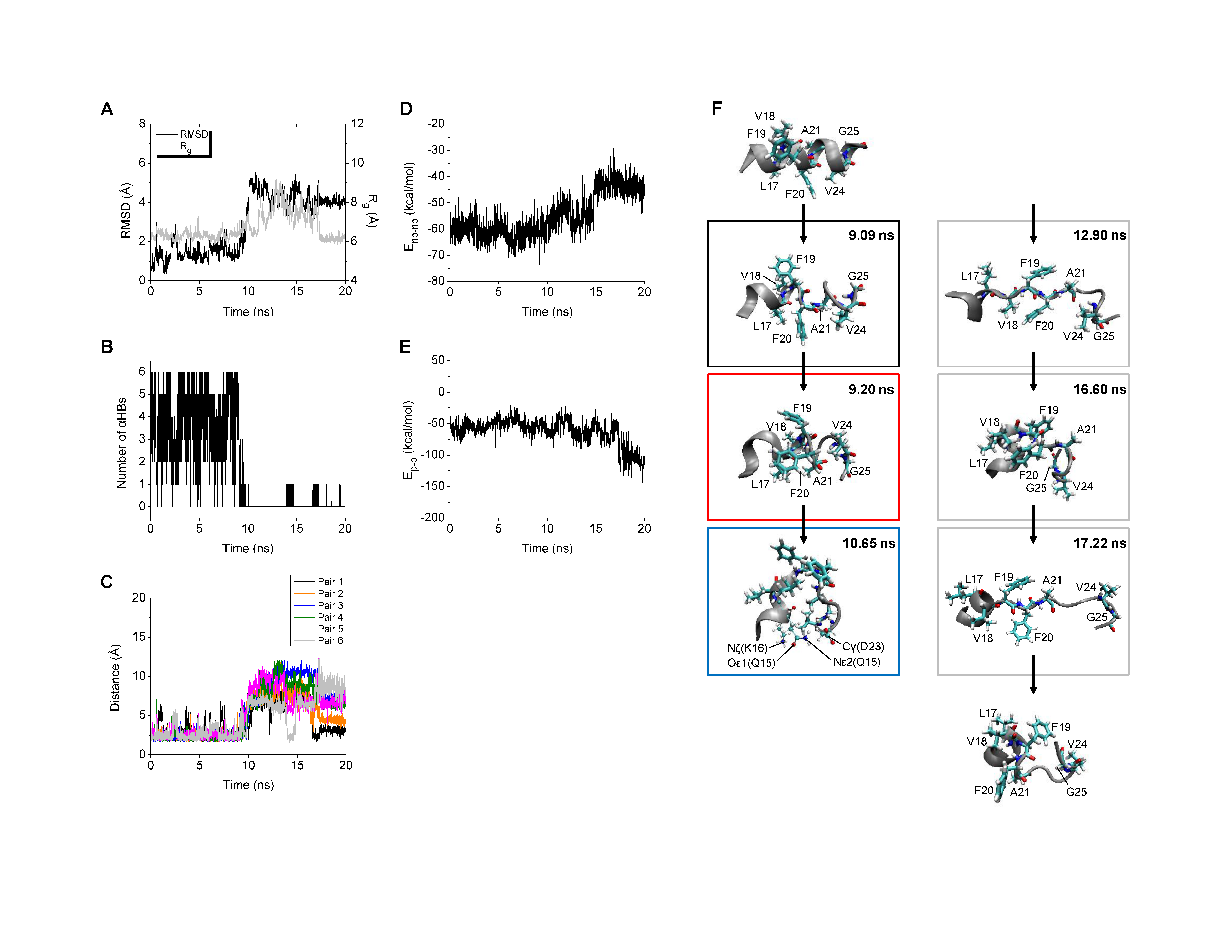

Supplement: Figure S4 — Structural and energetic changes of WT09. The RMSD and Rg (A), the number of αHBs (B), and the backbone O-HN distances of the αHB pairs 1–6 (C) calculated for the middle region (15–24) of the Aβ model are shown. The nonbonded interaction energies including E np-np (D) and E p-p (E) are also shown. The structure obtained at 9.09 ns when the number of αHBs starts to decrease, that obtained at 9.20 ns with the E np-np minimum (−73.54 kcal/mol), and that obtained at 10.65 ns with the notably low E p-p (−87.82 kcal/mol) are displayed in the black, red, and blue boxes, respectively (F). The structures obtained at 12.90, 16.60, and 17.22 ns with relatively large (9.19 Å), small (6.42 Å), and large (8.96 Å) Rg, respectively, are displayed from the top the bottom in the grey boxes. The initial energy-minimized structure and the structure obtained at 20.00 ns are also displayed at the top and the bottom, respectively. The positions of all the nonpolar residues (thick lines) and those of the polar residues (lines and balls) which are closely located are indicated. (TIF) [file pone.0017587.s004.tif]
